# Psychometric properties of Turkish Orthorexia Nervosa Inventory in a clinical adolescent sample

**DOI:** 10.1007/s40519-023-01601-z

**Published:** 2023-09-01

**Authors:** Bahadir Turan, Selman Yıldırım, Samiye Çilem Bilginer, Mehmet Akif Akıncı

**Affiliations:** 1 Department of Child and Adolescent Psychiatry, Faculty of Medicine, Karadeniz Technical University, 61000 Trabzon, Türkiye; 2 Graduate School of Natural and Applied Sciences, Ankara University, Interdisciplinary Artificial Intelligence Technology, Ankara, Türkiye; 3 Private Practice, Trabzon, Türkiye; 4 Department of Child and Adolescent Psychiatry, Faculty of Medicine, Ataturk University, Erzurum, Türkiye

**Keywords:** Orthorexia Nervosa Inventory, Adolescent, Validation, Orthorexia nervosa, Eating disorders

## Abstract

**Purpose:**

Orthorexic tendencies are increasingly prevalent among children and adolescents. This study set out to investigate the reliability and validity of the Turkish version of the Orthorexia Nervosa Inventory (ONI) in a clinical adolescent sample.

**Methods:**

266 adolescents aged 12–18 years, who applied to the Department of Child and Adolescents Psychiatry were included in the study. Participants completed sociodemographic data form, ONI, Eating Attitude Test, Revised Child Anxiety and Depression Scale-Child Version and ORTO-15.

**Results:**

The Cronbach’s alpha coefficient for the ONI reached 0.92, indicating very good internal consistency. Total factor scores and Cronbach alpha values for behaviors, impairments, and emotions were found to be 0.84, 0.84, and 0.83, respectively. The CFA performed supported the three-factor structure of the ONI obtained in the first sample. The minimum discrepancy per degree of freedom = 1.89 and the model generally fit well to the structure (RMSEA = 0.058, SRMR = 0.033, CFI = 0.92, TLI = 0.91).

**Discussion:**

This study has shown that the Turkish version of the ONI is a valid and reliable scale for specifying the tendency for Orthorexia Nervosa in a Turkish adolescent population. These findings contribute in several ways to our understanding of orthorexic tendencies and provide a basis for more concrete research data that can be obtained by using the ONI, which is a reliable scale in studies to be conducted among adolescents.

**Level of evidence:**

Level V, descriptive cross-sectional study.

## Introduction

Orthorexia nervosa (ON) is a relatively recently identified eating disorder characterized by an obsessive and extreme preoccupation with consuming only “pure” and “healthy” foods [1, 2]. Individuals with ON are consumed with a strict adherence to self-imposed dietary rules, often leading to malnutrition and social isolation. Symptoms may include a fixation on food preparation and purity, avoiding food groups deemed impure or unhealthy, and an exaggerated concern with the quality and origin of food [3–5]. The disorder has been linked to anxiety, depression, and obsessive–compulsive disorder, and can have serious physical and psychological consequences if left untreated [6].

Although ON is not officially recognized as a diagnosis in the DSM-V classification system [7], a growing number of researchers are working towards defining the diagnostic criteria for this condition [4, 5, 8]. Despite this, there remains a lack of consensus among researchers regarding the best approach to diagnosing ON. Several assessment scales have been developed, including the ORTO-15, which is the most frequently used, as well as the Terruel Orthorexia Scale, the Dusseldorf Orthorexia Scale, and the Orthorexia Nervosa Inventory (ONI) [9–12]. Despite the widespread use of the ORTO-15 in prevalence studies, there are significant variations in results even in studies with similar methodological approaches [13–15]. For instance, in one study with young adults, the prevalence of ON was found to be 10.9% using the ORTO-15, while in another study with a similar age group, the prevalence was found to be 60.3% using the same scale [16, 17]. The discrepancies in results and questions about the psychometric properties of the ORTO-15 have highlighted the need for the development of a new, more accurate screening tool or scale for ON. Such a tool would aid in the reliable and consistent diagnosis of this increasingly prevalent disorder.

The ONI, a scale recently developed by Oberle et al. (2021), has demonstrated superior internal consistency and test–retest reliability compared to previously used ON scales. Notably, the ONI includes a subscale specifically designed to measure physical impairments, which is considered a key diagnostic criterion for ON [12]. The prevalence of ON was found to be 4.5% in a study of participants aged 18–75 years with ONI, while it was found to be 4.2% in another study in young adults [12, 18]. In Türkiye, a study was conducted to assess the validity and reliability of ONI, yet it was limited to the adult population [19]. However, it is now well-established that eating disorders, including ON, are beginning at increasingly younger ages [3, 20, 21].

Therefore, although there have been many ON prevalence studies in adulthood, studies conducted in adolescents are crucial for enabling early screening and intervention. Given the reliable nature of the ONI scale and the necessity of conducting studies in the adolescent population, the aim of this study is to evaluate the validity and reliability of the ONI in Turkish adolescents.

## Methods

### Participants and procedure

This cross-sectional study was conducted between July and October 2022. Firstly, necessary permissions were obtained from the authors who developed ONI. In addition, the study was approved by Karadeniz Technical University Research Ethics Committee (2022–123). The required sample size for the study was calculated as at least 242 using the Tabachnick and Fidell (2007) method [22]. A total of 266 adolescents between the ages of 12–18 years, who were seeking treatment at the Department of Child and Adolescent Psychiatry in Karadeniz Technical University Hospital, were included to the study. Prior to the completion of the questionnaires, informed consent was obtained from both the participants and the parents of the participants. Participants completed various assessments, including the ONI, Eating Attitude Test, Revised Child Anxiety and Depression Scale-Child Version and ORTO-15, and a sociodemographic data form.

### Cross-cultural adaptation process

The translation and adaptation process was conducted by four child and adolescent psychiatrists who are native speakers of Turkish and fluent in English. First, the original scale was translated from English to Turkish using forward translation. Subsequently, the instruments were translated into English. Finally, the authors scrutinized the phraseology of both the English and Turkish versions and compared them with the original English version, with the objective of identifying uncertainties and correct inconsistencies.

### Measures

#### Sociodemographic data form

The researchers designed this form to obtain descriptive characteristics of the clinical sample. In the form, participants are asked to provide data on their ages, body mass indexes, genders, places of residence, parental education situations, family types, daily meal frequencies, and whether they use nutritional supplements.

#### Orthorexia Nervosa Inventory (ONI)

ONI is developed by Oberle et al. [12] to assess the symptomatology of ON. The scale consists of 24 items presented in a 4-point Likert-type format. It has shown high internal consistency, with a Cronbach's alpha coefficient of 0.94, indicating good reliability. The ONI is divided into three subscales, each targeting specific aspects of ON symptomatology:

Behavior Subscale (9 items): this subscale assesses the behavioral aspects of ON, focusing on the individual’s preoccupation with and adherence to rigid dietary rules and patterns. Respondents are asked to rate the frequency and intensity of their behaviors related to restrictive eating habits and obsession with healthy eating.

Impairment in Physical and Social Functioning Subscale (10 items): this subscale examines the impact of orthorexic behaviors on the individual's physical health and social interactions. It assesses how ON affects the person's ability to engage in normal daily activities, relationships, and social events.

Emotional Stress Subscale (5 items): this subscale evaluates the emotional and psychological consequences of orthorexic behaviors. It includes items related to anxiety, guilt, and distress associated with deviations from self-imposed dietary rules and the fear of consuming “unhealthy” foods.

Each subscale has shown good internal consistency, with Cronbach’s alpha coefficients ranging from 0.88 to 0.90. These coefficients suggest that the items within each subscale are highly interrelated and measure similar constructs. Higher scores on the ONI indicate more severe symptoms of ON, reflecting greater preoccupation with healthy eating, increased impairment in physical and social functioning, and elevated emotional stress related to dietary choices. A cut-off score of 72 on the scale is suggested to indicate a high risk for ON or the persistence of orthorexic symptoms. In summary, the ONI is a reliable and comprehensive tool for assessing orthorexia nervosa symptomatology, providing valuable insights into the behavioral, functional, and emotional aspects of this condition. Its subscales allow for a detailed understanding of the different dimensions of orthorexia nervosa, aiding in its identification and evaluation.

#### Revised Child Anxiety and Depression Scales—Child Version (RCADS-CV)

The RCADS-CV scale, developed by Chorpita et al. [23], is designed to measure anxiety disorders and symptoms of depression based on DSM-IV. The Turkish validity and reliability study of the 47-item scale was conducted by Görmez et al. [24] Subscale scores for Generalized Anxiety Disorder, Social Phobia, Separation Anxiety Disorder, Panic Disorder, Obsessive Compulsive Disorder and Major Depressive Disorder are obtained from the scale using a 4-point Likert-type scoring system. Forms that can be completed based on the notification of both parent and child are available. Our study determined a Cronbach's alpha coefficient of 0.78 for the RCADS-CV.

#### ORTO-15

The ORTO-15 is a self-assessment test composed of 15 items designed to evaluate the susceptibility to ON. Donini et al. (2005) developed the ORTO-15 by modifying the original scale originally designed by Bratman to include additional questions, resulting in a total of 15 items [2, 9]. For items 3, 4, 6, 7, 10, 11, 12, 14, and 15, responses are rated on a four-point Likert scale ranging from Always (scored as 1 point) to Never (scored as 4 points). Per the guidelines provided by the original authors, items 2, 5, 8, and 9 are subject to reverse scoring, where the values are assigned as follows: “always” is equivalent to 4, “often” to 3, “sometimes” to 2, and “never” to 1. For items 1 and 13, the scoring is as follows: “always” corresponds to 2, “often” to 4, “sometimes” to 3, and “never” to 1. Higher scores indicate a tendency towards normal eating behavior, while lower scores indicate the distinguishing criteria for ON. The scale has a minimum score of 15 and a maximum of 60, with a cut-off point of 40 points. ORTO-15 serves as a screening tool, and higher scores suggest a higher likelihood of orthorexia symptoms, but a formal diagnosis requires professional evaluation. The validity and reliability study of the ORTO-15 in Türkiye was conducted by Arusoğlu [25]. In the study, the Cronbach's alpha coefficient for the ORTO-15 was calculated as 0.85.

#### Eating Attitude Test-26 (EAT-26)

The Eating Attitude Test-26 (EAT-26) is a self-administered scale developed by Garner and Garfinkel [26] to assess the symptoms of anorexia nervosa. The 26 items on the EAT-26 are scored and summed, resulting in a score between 0 and 53. The cutoff point for the EAT-26 is set at 20 points. Individuals scoring 20 or above on the scale are classified as having abnormal eating behaviors, while those scoring below 20 are classified as having normal eating behaviors. Ergüney-Okumuş and Sertel-Berk conducted a Turkish adaptation of the EAT-26, which demonstrated high internal consistency (Cronbach’s alpha = 0.84) and test–retest stability (coefficient = 0.78) [27].

### Statistical analyses

The data obtained from the research were statistically analyzed using SPSS version 23.0 (The Statistical Package for Social Sciences). Normality of the distribution of continuous variables was checked through the Kolmogorov–Smirnov test, and independent sample *t*-tests and Chi-square analysis were used for intergroup comparisons. Continuous variables were presented as mean and standard deviations (mean ± SD), while categorical variables were presented as numbers and percentages (N, %).

To test the construct validity of the scale, confirmatory factor analysis (CFA) was conducted using AMOS 24.0 and LISREL software. Before CFA, outlier and normality analyzes were carried out, which involved examining skewness, kurtosis, Cook's and Leverage values. The parametric test conditions, item analyzes and Cronbach alpha values of the scale were also evaluated. Construct validity was assessed based on Cronbach's alpha values, which were analyzed before CFA. The two-stage process was followed for construct validity. The CFA analysis employed multiple fit indices (REMSEA, CFI, TLI) where CFI and TLI > 0.90 were considered acceptable limits, and CFI and TLI > 0.95 were regarded as perfect fit limits. For RMSEA, the acceptable limit was < 0.08, and the perfect fit limit was < 0.50 [28, 29]. Moreover, the Chi-square value in CFA was expected to be below 3 [29, 30]. The Pearson correlation analysis was performed to investigate the criterion-related validity of the scale, with the correlations between the Children's Anxiety and Depression Scale-Child Form (CASS), the ORTO-15 and the Eating Attitude Test-26 (EAT-26) scales evaluated.

## Results

As presented in Table 1, the study encompassed a total of 266 participants, of whom 66.5% were identified as female (*n* = 177), while the remaining 33.5% were identified as male (*n* = 89). The participants’ age range spanned from 12 to 18 years, with a mean age of 14.92 ± 1.58. Within the participant pool, 54.9% resided in rural areas (*n* = 146), while the remaining 45.1% resided in urban areas (*n* = 120).

**Table 1:** Sociodemographic characteristics of patients (*n* = 266)

Variables	N (%) or mean ± SD
Age (years)	14.92 ± 1.58
BMI (kg/m^2^)	21.88 ± 4.54
Gender	
Female	177 (66.5)
Male	89 (33.5)
Residence	
Rural area	146 (54.9)
Urban area	120 (45.1)
Family status	
Nuclear family	198(74.4)
Others	68(25.6)
Father’s education	
Illiterate	1 (0.4)
Primary education	99(39.2)
High School	87(32.7)
University	79 (29.7)
Mother’s education	
Illiterate	2(0.8)
Primary education	123(46.3)
High School	87(32.7)
University	54(20.3)
Number of children	
1 Child	28(10.5)
2 Children	106(39.8)
3 Children	89 (33.5)
4 or more	43( 16.2)
Number of meals	
1 or 2	107(40.2)
Between 2 and 4	139(52.3)
5 or more	20(7.5)
Nutritional supplement status	
Yes	38(14.3)
No	228(85.7)

The conducted confirmatory factor analysis (CFA) provided support for the three-factor structure of the ONI, as observed in the initial sample. The model demonstrated a satisfactory fit to the data, with a minimum discrepancy per degree of freedom of 1.89, indicating a reasonable level of fit. Moreover, various fit indices were examined, revealing favorable results for the model’s overall fit to the structure, including a root mean square error of approximation (RMSEA) value of 0.058, a standardized root mean square residual (SRMR) value of 0.033, a comparative fit index (CFI) value of 0.92, and a Tucker–Lewis index (TLI) value of 0.91, as depicted in Table 2.

**Table 2:** Model fit statistics

Index	CMIN/df	CFI	IFI	RMSEA	NNFI	SRMR
Perfect Fit	0–3	0.95 ≤ CFI ≤ 1.00	0.95 ≤ IFI ≤ 1.00	0.00 ≤ RMSEA ≤ 0.05	0.95 ≤ NNFI ≤ 1.00	0.00 ≤ SRMR ≤ 0.05
Good Fit	3–5	0.90 ≤ CFI ≤ 0.95	0.90 ≤ IFI ≤ 0.95	0.05 ≤ RMSEA ≤ 0.10	0.90 ≤ NNFI ≤ 0.95	0.05 ≤ SRMR ≤ 0.08
Study Findings	1.888	0.92	0.921	0.058	0.907	0.033

CMIN/df: discrepancy divided by degree of freedom, CFI: Comparative Fit Index, IFI: Incremental Fit Index, RMSEA: root mean square error of approximation, NNFI: Non-Normed Fit Index, SRMR: standardized root mean square residual

The reliability coefficient of each subscale and the entire scale was analyzed using Cronbach’s alpha coefficient. The ONI exhibited a Cronbach’s alpha coefficient of 0.92, indicating a high level of internal consistency. Furthermore, the total factor scores and Cronbach’s alpha values for behaviors, impairments, and emotions were determined to be 0.84, 0.84, and 0.83, respectively, demonstrating good reliability within these specific dimensions.

The standardized factor loadings for items in the ONI were analyzed across three dimensions: Behaviors, Emotions, and Impairments, as presented in Table 3. The analysis revealed notable factor loadings, with ONI-18 demonstrating a strong association with Impairments (0.81), ONI-6 exhibiting a robust association with Emotions (0.81), and ONI-2 displaying a moderate association with Behaviors (0.39). These findings provide valuable insights into the intricate relationships between specific items and their corresponding factors within the ONI assessment tool.

**Table 3:** Standardized factor loadings

Items	ONI behaviors	ONI emotions	ONI impairments
*ONI-2*	0.39		
*ONI-4*	0.65		
*ONI-6*	0.62		
*ONI-8*	0.61		
*ONI-11*	0.68		
*ONI-15*	0.66		
*ONI-17*	0.45		
*ONI-18*	0.81		
*ONI-22*	0.55		
*ONI-1*		0.77	
*ONI-9*		0.76	
*ONI-13*		0.81	
*ONI-21*		0.74	
*ONI-23*		0.54	
*ONI-3*			0.33
*ONI-5*			0.54
*ONI-7*			0.70
*ONI-10*			0.44
*ONI-12*			0.80
*ONI-14*			0.50
*ONI-16*			0.43
*ONI-19*			0.63
*ONI-20*			0.57
*ONI-24*			0.69

Table 4 reveals significant correlations between the ONI and other variables. The ONI total scores exhibited strong positive correlations with ONI Behaviors (*r* = 0.867, *p* = 0.001), ONI Emotions (*r* = 0.868, *p* = 0.001), and ONI Impairments (*r* = 0.887, *p* = 0.001), indicating a robust relationship between these dimensions. Additionally, the ONI Total scores demonstrated moderate positive correlations with BMI (*r* = 0.183, *p* = 0.004), suggesting a link between orthorexic tendencies and body mass index. Furthermore, the ONI total scores showed a moderate negative correlation with ORTO-15 (*r* = − 0.285, *p* = 0.001), and a moderate positive correlation with EAT-26 (*r* = 0.542, *p* = 0.001), suggesting a connection between orthorexic tendencies and disordered eating attitudes. These findings, as presented in Table 4, provide important insights into the relationships between the ONI and various constructs, shedding light on the construct validity and associations of the ONI with other measures.

**Table 4:** Correlations between the ONI and other variables

	ONI total	ONI behaviors	ONI emotions	ONI impairments
ONI total	–	0.867*	0.868*	0.887*
ONI behaviors	0.867*	–	0.649*	0.604*
ONI emotions	0.868*	0.649*	–	0.694*
ONI impairments	0.887*	0.604*	0.694*	–
BMI	0.183*	0.085	0.220*	0.188*
EAT-26	0.542*	0.329*	0.571*	0.544*
ORTO-15	− 0.285*	− 0.255*	− 0.296*	− 0.210*
RCADS-CV total ANXIETY	0.354*	0.143	0.412*	0.397*
RCADS-CV total internalizing	0.348*	0.136	0.407*	0.394*

ONI: Orthorexia Nervosa Inventory, BMI: body mass index, EAT-26: Eating Attitude Test-26

*Correlation is significant at the 0.01 level (2-tailed)

Table 5 presents the relationship between the subdimensions of the ONI and gender. The mean scores and standard deviations for girls and boys are reported for the total ONI score, ONI Behaviors, ONI Emotions, and ONI Impairments. The analysis revealed significant differences between genders, with girls showing higher scores on ONI Impairments (*M* = 8.03, SD = 3.63) compared to boys (*M* = 6.73, SD = 2.40), t(245) = 3.50, *p* < 0.001. Additionally, girls exhibited higher scores on ONI Emotions (*M* = 13.90, SD = 5.03) compared to boys (*M* = 12.69, SD = 4.09), t(212) = 2.12, *p* = 0.035.

**Table 5:** Relationship between ONI subscales and genders

	Girl		Boy				
	M	SD	M	SD	df	*t*	*p*
ONI total	35.59	11.96	33.39	8.91	227	1.69	0.09
ONI behaviors	13.65	4.88	12.99	3.89	215	0.61	0.54
ONI emotions	8.03	3.63	6.73	2.40	245	3.50	0.001*
ONI impairments	13.90	5.03	12.69	4.09	212	2.12	0.035*

*ONI* Orthorexia Nervosa Inventory

^*^p < 0.050

## Discussion

This study aimed to investigate the psychometric properties of the Turkish version of the ONI in a clinical sample of adolescents, representing the first examination of the validity and reliability of an ON scale specifically in adolescent population. The results revealed favorable internal consistency, as indicated by a Cronbach's alpha coefficient of 0.92, consistent with previous studies [12, 19]. Furthermore, the sub-factors of “Behaviors”, “Impairments”, and “Emotions” demonstrated Cronbach’s alpha values of 0.84, 0.84, and 0.83, respectively. These findings support the reliable utilization of the Turkish version of the ONI scale among adolescents. In a previous study by Seda et al. [19] investigating the psychometric properties of the Turkish version of the ONI scale in adults, the internal consistency coefficient was reported as 0.91, with Cronbach’s alpha values ranging from 0.81 to 0.84 for the three sub-factors. The original scale developed by Oberle et al. [12] demonstrated an internal consistency coefficient of 0.94.

Investigating the relationship between ON symptoms and various conditions is of utmost importance to elucidate whether this condition represents a distinct disorder or simply a variant within the spectrum of normal behaviors. The correlation matrix presented herein sheds light on the associations between the subscales of the ONI and a range of pertinent variables. The results of this study suggest that ON symptoms, as assessed by the ONI, exhibit connections with eating disorder symptoms, orthorexia tendencies, anxiety, internalizing symptoms, and, to a lesser degree, body mass index (BMI). It is worth noting that the existing literature lacks consensus regarding the comorbidities commonly observed alongside ON [31–36]. These observed associations offer invaluable insights into the intricate interplay between ON and related psychological factors. By enhancing our comprehension of this disorder, they contribute significantly to the ongoing pursuit of unraveling its clinical nature and distinguishing it from normative behaviors.

The present study revealed significant correlations between ONI subscale scores and EAT-26 scores, consistent with the findings of Oberle et al. (2021), who also reported a positive association between EAT-26 scores and ONI scale scores, highlighting the role of eating attitudes as a prominent predictor of ON symptomatology [12]. These findings align with existing literature and provide further evidence for the progressive nature of ON in relation to disordered eating attitudes [12, 37–39]. The study presents the relationship between ONI subscales and gender, indicating that while there were no significant differences in the total ONI score and ONI Behaviors subscale, girls exhibited higher levels of orthorexia-related emotions and impairments compared to boys, providing valuable insights into the gender-specific aspects of ON symptoms. The findings from this analysis contribute to our understanding of how ON may manifest differently in boys and girls. The absence of significant gender differences in the total ONI score and ONI Behaviors subscale suggests that the overall severity of orthorexia symptoms and the behavioral aspects of the disorder may not differ significantly between boys and girls in this sample. However, the significant gender differences observed in the ONI Emotions and ONI Impairments subscales highlight distinct patterns in the emotional and functional consequences of ON. The higher levels of orthorexia-related emotions reported by girls may indicate that they experience greater psychological distress and preoccupation with their eating behaviors [40, 41]. On the other hand, the higher levels of impairments reported by girls suggest that they may experience more functional limitations and difficulties in their daily lives due to orthorexia-related behaviors and beliefs. The fact that ONI total scores indicating the risk of ON in this study did not differ between genders was consistent with the existing literature [19, 42].

The study has several limitations that should be acknowledged. Firstly, the data were collected from adolescents seeking treatment at a child and adolescent psychiatry outpatient clinic, which may introduce selection bias and limit the generalizability of the findings to the broader adolescent population. Future studies should consider including community samples to obtain a more representative understanding of ONI scores and comorbidities. Secondly, the study was conducted at a single center in a university hospital, which may limit the generalizability of the results to other settings and cultural contexts. Replicating the study in multiple centers and diverse cultural contexts would provide a more robust understanding of the phenomenon. Thirdly, the majority of the research participants were girls, resulting in an imbalance in gender representation. Conducting studies with a more balanced distribution of male and female participants would yield stronger and more comprehensive data regarding the relationship between ON and gender. Fourthly, the assessment of ON relied solely on self-report measures. To enhance the validity and reliability of the findings, future research could consider incorporating scales that involve parent or teacher reporting, allowing for a more objective evaluation and comparison of results. These limitations should be taken into consideration when interpreting the findings of the study and highlight the need for further research to address these limitations and expand our understanding of ON in diverse populations and assessment approaches.

This study has demonstrated the validity and reliability of the Turkish version of the ONI as a robust scale for assessing the tendency towards ON in Turkish adolescents. Notably, the ONI stands out as the first orthorexia scale to incorporate items assessing physical impairments, a significant component of the disorder. These findings contribute substantially to our understanding of orthorexic tendencies and provide a solid foundation for future research utilizing the ONI as a reliable instrument in adolescent studies, yielding more definitive and comprehensive data in this population.

### What is already known on this subject?

The ONI, developed by Oberle et al., distinguishes itself with both its impairment evaluation and stronger psychometric properties compared to other scales. A validity and reliability study of the ONI has been conducted in Türkiye, but it was exclusively performed with adult samples. To date, there has been no investigation into the Turkish validity and reliability of the ONI among adolescents.

### What does this study add?

This study has demonstrated that the Turkish version of the ONI is a valid and reliable scale for assessing the tendency towards Orthorexia Nervosa in the Turkish adolescent population. Furthermore, its implementation will serve as a valuable contribution to future research on orthorexia nervosa among adolescents, as this age group represents a vulnerable population concerning the development of eating disorders.

## Data Availability

You can contact the corresponding author for any questions and requests related to the data.
